# Assessing the accuracy of [^18^F]PSMA-1007 PET/CT for primary staging of lymph node metastases in intermediate- and high-risk prostate cancer patients

**DOI:** 10.1186/s13550-022-00918-7

**Published:** 2022-08-09

**Authors:** Jacob Ingvar, Erland Hvittfeldt, Elin Trägårdh, Athanasios Simoulis, Anders Bjartell

**Affiliations:** 1 Department of Urology, Skåne University Hospital, Malmö, Sweden; 2 Department of Translational Medicine, Faculty of Medicine, Lund University, Jan Waldenströmsgata 5, 205 02 Malmö, Sweden; 3 Clinical Physiology and Nuclear Medicine, Skåne University Hospital, Malmö, Sweden; 4 Wallenberg Centre for Molecular Medicine, Lund University, Lund, Sweden; 5 Department of Pathology, Skåne University Hospital and Lund University, Malmö, Sweden

**Keywords:** Lymph node dissection, Metastases, PET/CT, Prostate cancer, Robotic surgery, Staging

## Abstract

**Background:**

[^18^F]PSMA-1007 is a promising tracer for integrated positron emission tomography and computed tomography (PET/CT).

**Objective:**

Our aim was to assess the diagnostic accuracy of [^18^F]PSMA-1007 PET/CT for primary staging of lymph node metastasis before robotic-assisted laparoscopy (RALP) with extended lymph node dissection (ePLND).

**Design, Setting and Participants:**

The study was a retrospective cohort in a tertiary referral center. Men with prostate cancer that underwent surgical treatment for intermediate- or high-risk prostate cancer between May 2019 and August 2021 were included.

**Interventions:**

[^18^F]PSMA-1007 PET/CT for initial staging followed by RALP and ePLND.

**Outcome measurements and statistical analyses:**

Sensitivity and specificity were calculated both for the entire cohort and for patients with lymph node metastasis ≥ 3 mm. Positive (PPV) and negative (NPV) predictive values were calculated.

**Results and limitations:**

Among 104 patients included in the analyses, 26 patients had lymph node metastasis based on pathology reporting and metastases were ≥ 3 mm in size in 13 of the cases (50%). In the entire cohort, the sensitivity and specificity of [^18^F]PSMA-1007 were 26.9% (95% confidence interval (CI); 11.6–47.8) and 96.2% (95% CI; 89.2–99.2), respectively. The sensitivity and specificity of [^18^F]PSMA-1007 to detect a lymph node metastasis ≥ 3 mm on PET/CT were 53.8% (95% CI; 25.1–80.8) and 96.7% (95% CI; 90.7–99.3), respectively. PPV was 70% and NPV 93.6%.

**Conclusions:**

In primary staging of intermediate- and high-risk prostate cancer, [^18^F]PSMA-1007 PET/CT is highly specific for prediction of lymph node metastases, but the sensitivity for detection of metastases smaller than 3 mm is limited. Based on our results, [^18^F]PSMA-1007 PET/CT cannot completely replace ePLND.

**Patient summary:**

This study investigated the use of an imaging method based on a prostate antigen-specific radiopharmaceutical tracer to detect lymph node prostate cancer metastasis. We found that it is unreliable to discover small metastasis.

## Introduction

Prostate cancer is the most common cancer form in developed countries, and the incidence is increasing [1]. A significant proportion of patients diagnosed with localized prostate cancer is successfully managed with radical prostatectomy or radiotherapy. Accurate assessment of tumor stage, regional lymph node involvement and distant metastasis are essential to recommend proper treatment.

Conventional imaging, such as computed tomography (CT), magnetic resonance imaging (MRI), and bone scintigraphy (BS), have shown limited sensitivity to detect prostate cancer metastasis [2, 3]. Molecular imaging, using different radiopharmaceuticals, has shown promising results in visualizing the presence and extent of prostate cancer lesions [4, 5]. Radiolabeled choline positron emission tomography/computed tomography (choline PET/CT) was previously used to identify lymph node metastasis, but studies have shown a poor accuracy [5–7]. Ligands targeting prostate-specific membrane antigen (PSMA) have been introduced in PET/CT for localization of recurrent disease and more recently for primary staging of high-risk tumors. Several tracers have been developed for PSMA PET/CT. Studies have shown promising results with [^68^ Ga]Ga-PSMA-11 for both staging and disease recurrence [4, 8, 9]. In a recent study by Fendler et al. [10], a [^68^ Ga]Ga-PSMA-11 PET/CT scan changed management in more than 50% of cases with biochemical recurrence (BCR). Similar results were shown in another study by Sonni et al. in 2020 [11]. [^18^F]PSMA-1007 is a radiopharmaceutical which has not been evaluated to the same degree as [^68^ Ga]Ga-PSMA-11. It has the advantage of a longer half-life and offers a low urinary clearance compared to [^68^ Ga]Ga-PSMA-11 PET/CT. However, a previous study by Vollnberg et al. showed that [^18^F]PSMA-1007 PET/CT scans often present focal unspecific bone uptake, that should be interpreted carefully to avoid over-staging [12].

A head-to-head comparison by Kuten et al. [13] showed that both [^18^F]PSMA-1007 and [^68^ Ga]Ga-PSMA-11 identified all lesions in the prostate in intermediate- or high-risk prostate cancer patients at staging. Similarly, Hoberück et al. [14] found no difference in staging accuracy between the two tracers.

The aim of this study was to evaluate the accuracy of [^18^F]PSMA-1007 in lymph node staging in intermediate- and high-risk prostate cancer, using histopathology after extended pelvic lymph node dissection (ePLND) as reference method. We investigated a consecutive, retrospective cohort in a tertiary referral center.

## Material and methods

All patients who underwent primary staging of intermediate- or high-risk prostate cancer with [^18^F]PSMA-1007 PET/CT followed by robotic-assisted laparoscopic prostatectomy (RALP), with extended pelvic lymph node dissection (ePLND) from May 2019 until August 2021, were eligible to be included in the study. The study was approved by the Regional Ethics Committee in Lund (nr 2016/417, 2018/753), and all patients signed an informed consent. Data extracted from medical records included age, date of diagnosis, treatment modality, PSA, Gleason score, results of PSMA PET/CT and details from pathology reports. Patients were classified as intermediate- or high risk based on the EAU guidelines risk groups. Surgery was performed by experienced surgeons at Skåne University Hospital in Malmö using a detailed template. Bilateral ePLND was defined as removal of the tissue from the bifurcation of the common iliac artery and distally along the external iliac vessels, above the internal iliac artery and deep in the obturator fossa. Tissue from the left and right side was sent separately for histopathological examination. ePLND was performed in conjunction with RALP in every case.

### PET/CT imaging

Patients were administered 4 MBq/kg [^18^F]PSMA-1007 (median 4.0, range 3.7–6.7) through intravenous bolus injection. A head-to-knee PET/CT scan was performed using GE Discovery MI PET-CT systems (Discovery MI; GE Healthcare, Milwaukee, WI, USA) 120 min (median 120, range 115–130) after administration of the radiopharmaceutical [15]. No forced diuresis was applied as [^18^F]PSMA-1007 is mainly eliminated through bile. Acquisition time was 2 min/bed position (3 min when BMI > 40). The PET/CT-system has a 128-slice CT. The Q.Clear reconstruction algorithm was used, including time-of-flight, point spread function and CT-based attenuation correction with a 256 × 256 matrix (pixel size, 2.7 × 2.7 mm^2^, slice thickness, 2.8 mm [16]. The noise-regularization parameter (β) was set to 800 [17]. An adaptive statistical iterative reconstruction technique was used for the CT images.

PET/CT images were evaluated for lymph node metastases by a nuclear medicine physician with > 5 years' experience of whole-body PET/CT. Evaluation was done blinded and compared with a previous evaluation done for clinical purposes. If there was disagreement between the evaluations or if the clinical evaluation was inconclusive a third evaluation was performed by a nuclear medicine specialist with > 10 years' experience of PET/CT. Lymph nodes were graded 1–5 according to the E-PSMA reader confidence scale [18]. Grade 1–2 lymph nodes were considered non-pathological, while grade 4–5 were considered pathological. Grade 3 includes “faint uptake in a site typical for prostate cancer.” We considered lymph node uptake clearly visible (with standardized uptake value threshold 0–10) but clearly below spleen as faint. Grade 3 was considered pathological when deviating from known patterns of unspecific uptake (mainly faint symmetric uptake along the external iliac vessels or intense uptake in thorax or axilla). Maximum standardized uptake value (SUVmax) calculated by body weight was measured in the prostate.

### Histopathology

Lymph node specimens from the left and right side were examined in a blinded fashion by experienced pathologists at Skåne University Hospital using routine methods. According to standard of practice in Sweden, the presence and size of metastases and the total number of lymph nodes extracted from each side were reported in detail.

### Statistical analysis

Statistical analysis was performed using STATA (StataCorp. 2015. Stata Statistical Software: Release 14. College Station, TX: StataCorp LP). Sensitivity, specificity, positive predictive value (PPV) and negative predictive value (NPV) including 95% confidence intervals (CI) were calculated for [^18^F]PSMA-1007 PET/CT scan using the histology results after ePLND as gold standard. Two patient-based scenarios were analyzed: firstly, the ability of [^18^F]PSMA-1007 PET/CT scanning to detect the presence of lymph node metastasis of any size in a patient, and secondly the ability to detect lymph node metastasis ≥ 3 mm in diameter. The size of the metastasis and not the lymph node itself was reported. Considering all lymph nodes obtained at ePLND as separate observations, the ability of [^18^F]PSMA-1007 PET/CT scan to detect the presence of metastasis of any size in lymph nodes was analyzed (lesion-based analyses). The Mann–Whitney U-test was used for comparison of SUVmax in the prostate. A *p* value less than 0.05 was considered statistically significant.

## Results

We evaluated a consecutive series of 106 intermediate- or high-risk prostate cancer patients who were evaluated with [^18^F]PSMA-1007 PET/CT prior to RALP with ePLND between May 2019 and August 2021. Two patients were excluded since a follow-up [^18^F]PSMA-1007 PET/CT was positive for lymph nodes on the exact location of the initial [^18^F]PSMA-1007 PET/CT, suggesting these lymph nodes were not removed during ePLND. After exclusions, 104 patients remained for final analysis. Patient characteristics are summarized in Table 1. In total, 2519 lymph nodes were removed by ePLND and prostate cancer metastases were found by histopathological examination in 41 lymph nodes from 26 patients (Table 2).

**Table 1:** Characteristics of patients in final analyses

Patient and tumor characteristics	*N* (%)
Total number of patients	104
Age (mean, range)	66.0 (42–76)
PSA at diagnosis ng/mL	
Mean	12.6 (2.2–86)
< 10	58 (55)
10–19.9	28 (27)
> 20	19 (18)
Clinical tumor stage	
T1	48 (46)
T2	47 (45)
T3	10 (9)
EAU risk group, *n* (%)	
Low	0 (0)
Intermediate	24 (23)
High	80 (77)
ISUP grade at biopsy	
1	1 (1)
2	11 (10)
3	30 (29)
4	28 (27)
5	35 (33)

**Table 2:** Histopathology reported lymph nodes removed at ePLND

Total number of lymph nodes removed from 104 patients	*n* = 2519
Total number of lymph nodes with histologically confirmed metastasis (%)	*n* = 41 (1.6%)
Mean number of lymph nodes removed per patient (range)	24.2(6–48)
Median number of lymph nodes removed	23
Lymph node metastasis ≥ 3 mm that were positive on [^18^F]PSMA-1007 PET/CT	7
Lymph node metastasis < 3 mm that were positive on [^18^F]PSMA-1007 PET/CT	0

Lymph node metastasis was detected pre-operatively by [^18^F]PSMA-1007 in 7 of these 26 patients resulting in a sensitivity of 26.9% (95% CI; 11.6–47.8) and a specificity of 96.2% (95% CI; 89.2–99.2) (Table 3). PPV and NPV were 70% and 79.8%, respectively (Table 3).

**Table 3:** Lymph node status from histopathology report (PAD) and by [^18^F]PSMA-1007 PET/CT for all patients and if gold standard was lymph node ≥ 3 mm (number of patients)

	PAD positive	PAD negative	Total
All patients			
PSMA PET/CT positive	7	3	10
PSMA PET/CT negative	19	75	94
Total	26	78	104
Lymph nodes ≥ 3 mm			
PSMA PET/CT positive	7	3	10
PSMA PET/CT negative	6	88	94
Total	13	91	104

For lymph nodes metastases ≥ 3 mm in diameter, [^18^F]PSMA-1007 PET/CT detected 7 out of 13 metastases, resulting in a sensitivity and specificity of 53.8% (95% CI; 25.1–80.8) and 96.7% (95% CI; 90.7–99.3), respectively. PPV and NPV for detecting lymph nodes ≥ 3 mm were 70% and 93.6%, respectively (Table 3). The area under curve (AUC) in receiver operating characteristics (ROC) analysis for [^18^F]PSMA-1007 PET/CT to detect lymph node metastases larger than 3 mm in size was 0.75 (95% CI; 0.61–0.89) compared to 0.62 (95% CI; 0.53–0.71) for metastases of any size.

In high-risk prostate cancers, the sensitivity to detect lymph node metastasis of any size with [^18^F]PSMA-1007 PET/CT pre-operatively was 35% (95% CI: 15.4–59.2) (Tables 4 and 5). Only one patient had a positive [^18^F]PSMA-1007 PET/CT in the intermediate risk group. The specificity was high in both groups; 96.7% (95% CI; 88.5–99.6) for high-risk and 94.4% (95% CI; 72.7–99.9) for intermediate prostate cancers. In the high-risk group, PPV and NPV for detecting lymph nodes were 77.8% and 81.7%, respectively, and AUC ROC was 0.66 (95% CI; 0.55–0.77).

**Table 4:** Lymph node status by PSMA and by histology report (PAD) for intermediate and high risk

	PAD positive	PAD negative	Total
Intermediate risk			
PSMA positive	0	1	1
PSMA negative	6	17	23
High risk			
PSMA positive	7	2	9
PSMA negative	13	58	71
Total	26	78	104

**Table 5:** Sensitivity, specificity, positive predictive value (PPV) and negative predictive value (NPV) for prediction of lymph node metastases by PET/CT (95% confidence interval) with histopathology report as reference method based on risk groups

	Intermediate risk	High risk
Sensitivity	0 (0–45.9)	35 (15.4–59.2)
Specificity	94.4 (72.7–99.9)	96.7 (88.5–99.6)
PPV	0	77.8
NPV	73.9	81.7

### Quantification of tracer uptake in lymph nodes and in the prostate

**Fig. 1:**
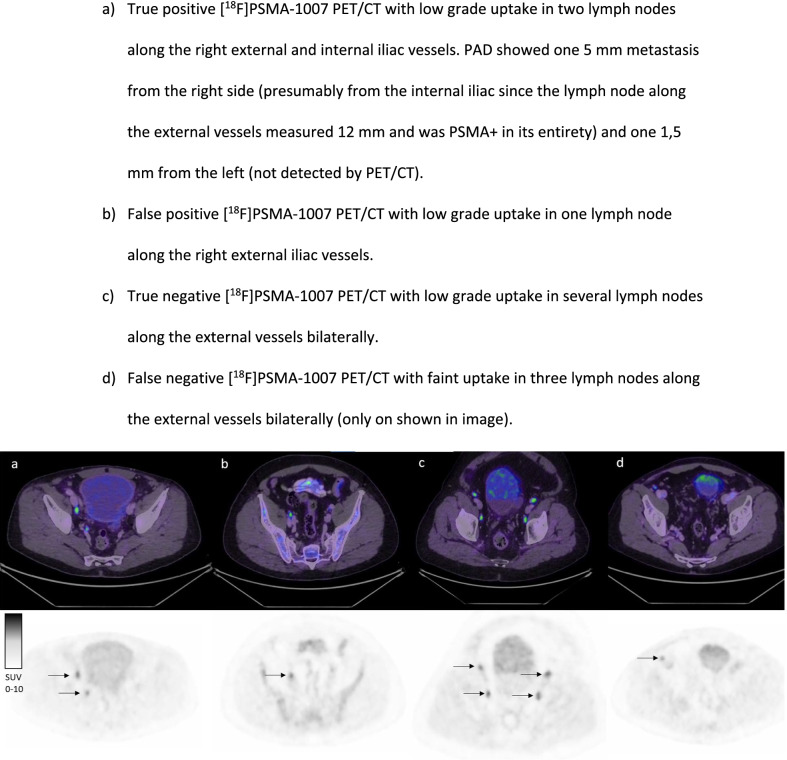
Examples of true and false positives and negatives with [^18^F]PSMA-1007 PET/CT. **a** True positive [^18^F]PSMA-1007 PET/CT with low grade uptake in two lymph nodes along the right external and internal iliac vessels. PAD showed one 5 mm metastasis from the right side (presumably from the internal iliac since the lymph node along the external vessels measured 12 mm and was PSMA + in its entirety) and one 1,5 mm from the left (not detected by PET/CT). **b** False positive [^18^F]PSMA-1007 PET/CT with low grade uptake in one lymph node along the right external iliac vessels. **c** True negative [^18^F]PSMA-1007 PET/CT with low grade uptake in several lymph nodes along the external vessels bilaterally. **d** False negative [.^18^F]PSMA-1007 PET/CT with faint uptake in three lymph nodes along the external vessels bilaterally (only on shown in image)

Three lymph nodes were classified as false positives on [^18^F]PSMA-1007 PET/CT using histopathology as gold standard. They all displayed faint uptake in non-enlarged lymph nodes asymmetrically distributed along the external iliac vessels. All true positives showed moderate (close to or above spleen) or higher uptake on [^18^F]PSMA-1007 PET/CT along the external iliac vessels or faint or higher uptake along the internal iliac vessels (Fig. 1).

There was no significant difference in SUVmax in the prostate between true positives (median 20.2, range 8.0–53.9, *n* = 7) and false negatives (median 17.0, range 5.2–47.5, *n* = 19), *p* = 0.59.

A sensitivity analysis was performed to investigate if sensitivity and specificity were affected if all grade 3 lymph nodes were either regarded as pathological or non-pathological. Regarding all grade 3 lymph nodes as pathological gave a sensitivity of 31%, specificity of 78% and AUC of 0.55, compared to a sensitivity of 17%, specificity of 100% and AUC of 0.56 when all grade 3 lymph nodes were classified as non-pathological.

## Discussion

In this study of intermediate- and high-risk prostate cancer patients evaluated with [^18^F]PSMA-1007 PET/CT pre-operatively it was found that the sensitivity for detection of lymph node metastasis of any size was 26.9% and 53.8% for lymph node metastasis > 3 mm in size. However, [^18^F]PSMA-1007 PET/CT was found to be highly specific, ruling out metastasis in 96.2% of patient with no metastases on histopathological examination.

Correct staging of prostate cancer is essential to select the most appropriate treatment for every patient. Conventional imaging such as CT, MRI, ^18^F-choline PET/CT and BS has shown poor accuracy in both detecting and ruling out metastases in the lymph nodes [2, 7]. In the EAU guidelines for prostate cancer, lymph node dissection is recommended for staging of prostate cancer in some patients based on risk prediction by nomograms; however, the procedure is associated with significant side effects, and no survival benefit has been demonstrated [19–21].

There is a need for improvement in prostate cancer staging, and different tracers used in PET/CT have been shown to provide additive value. [^68^ Ga]Ga-PSMA-11 has been investigated in several studies with promising results for both staging and recurrence detection [4, 8–10]. In a recent study by Moreira et al. [22], the sensitivity and specificity for lymph node metastases were 75% and 90%, respectively. Due to the longer half-life and lower urinary clearance, presumably causing less artifacts in PET/CT, [^18^F]PSMA-1007 PET/CT might further improve staging, but the method has not yet been sufficiently investigated. Prior studies evaluating the role of [^18^F]PSMA-1007 PET/CT in primary staging of prostate cancer have reported on limited patient cohorts [23, 24]. Giesel et al. [23] found a high sensitivity of 94.7%, and however, their results were based on only eight patients. Similarly, Kesch et al. [24] analyzed ten patients and found a sensitivity and specificity of 71% and 81%, respectively. To the best of our knowledge, only three larger studies on prostate cancer staging using [^18^F]PSMA-1007 PET/CT have been published. In line with the results of our study, Meijer et al. [25] analyzed 757 patients and found a sensitivity of 38% and a specificity of 94%. However, they used three different tracers, [^68^ Ga]Ga-PSMA-11, ^18^F-DCFPyL and ^1^[^18^F]PSMA-1007, which makes it difficult to compare with our present results. In a study by Hermsen et al. [26], 99 men were evaluated with a sensitivity of 53.3% and a specificity of 89.9% which is in line with our study. In the third study by Sprute et al. [27], the accuracy of [^18^F]PSMA-1007 PET/CT in lymph node staging was investigated in 96 prostate cancer patients. They found a significantly higher sensitivity of 73.5% and somewhat higher specificity of 99.4% than in our study. The differences between our results and those reported by Sprute et al. can to some extent be explained by patient selection. While our study included only patients that had been staged with [^18^F]PSMA-1007 PET/CT prior to ePLND performed simultaneously with RALP, Sprute et al. included [^18^F]PSMA-1007 PET/CT scannings performed both for staging and salvage ePLND. For patients with BCR, another tracer, [^18^F]-rhPSMA-7, has also been investigated [28–30] showing a high detection efficacy. In a study on 58 patients by Kroenke et al. [31] using this tracer, the sensitivity and specificity for detecting lymph node metastasis prior to salvage surgery were 72.2% and 92.5%, respectively. These results are difficult to compare to the results of our study due to the diverse patient base. On another note, a prior study analyzing pitfalls of PSMA-PET/CT found that PSMA-ligand uptake in benign lesions was considerably higher with [^18^F]PSMA-1007 compared to [^68^ Ga]Ga-PSMA-11 [32]. Overall, due to the lack of head-to-head studies, it is still unclear if one PSMA-tracer offers significant advantages over the others in prostate cancer staging.

Our subanalysis of the metastasis size showed a significantly higher sensitivity and specificity with a larger metastasis size. This was also seen in Sprute et al. where the detection of nodes more than 3 mm in size the sensitivity and specificity was 85.9% and 99.5%, respectively. It is unclear if Sprute et al. measured the size of the metastasis or the size of the lymph node, which makes the results difficult to compare. Nevertheless, in line with the study by Sprute et al., as well as studies of [^68^ Ga]Ga-PSMA-11, we found that [^18^F]PSMA-1007 PET/CT has a lower detection rate for small lymph node metastasis [27, 33]. In fact, no metastasis < 3 mm in size was identified on [^18^F]PSMA-1007 PET/CT in our study.

We attempted to analyze the benefit of [^18^F]PSMA-1007 PET/CT in different risk categories, according to the EAU risk groups for prostate cancer patients. Due to low numbers in the intermediate group, only one patient had a positive [^18^F]PSMA-1007 PET/CT, which makes it impossible to draw any conclusion. Regarding the interpretation of [^18^F]PSMA-1007 PET/CT scans we found that faint tracer uptake along the external iliac vessels is unspecific and should be interpreted cautiously. False negative findings were not associated with lower SUVmax in the prostate compared to true positives. Again, the low number of true positive cases makes our results uncertain.

Our study utilizes PET scanners with silicon photomultiplier technology and the Q.Clear reconstruction algorithm. Both these technologies can improve image quality and potentially lesion detection [34–36]. In prostate cancer, this has, to our knowledge, only been studied in BCR with promising results [37, 38]. It is possible that a lower sensitivity would have been found with conventional PET scanners.

The strengths of this study are the population-based design, the meticulous information collection, and the blinded assessment of [^18^F]PSMA-1007 PET/CT scans by one nuclear medicine physician. The main limitations are the retrospective design of the study and the limited number of patients.

## Conclusion

We found [^18^F]PSMA-1007 PET/CT to be highly specific for prediction of lymph node metastases in patients with intermediate- or high-risk prostate cancer, but with low sensitivity for detection of metastatic lesions smaller than 3 mm in size. Currently, [^18^F]PSMA-1007 PET/CT cannot completely replace staging with ePLND in patients with no signs of distant metastases.

## Data Availability

The datasets from the study can be made available from the corresponding author on reasonable request.
